# Supplementary material for: Voltammetric Detection of Glucose—The Electrochemical Behavior of the Copper Oxide Materials with Well-Defined Facets
Source: Sensors (Basel). 2022 Jun 24;22(13):4783. doi: 10.3390/s22134783 (PMC9269370; doi:10.3390/s22134783)
Supplement: Supplementary file 1 [file sensors-22-04783-s001.zip › sensors-1733878-supplementary.pdf]

Suplementary Materials

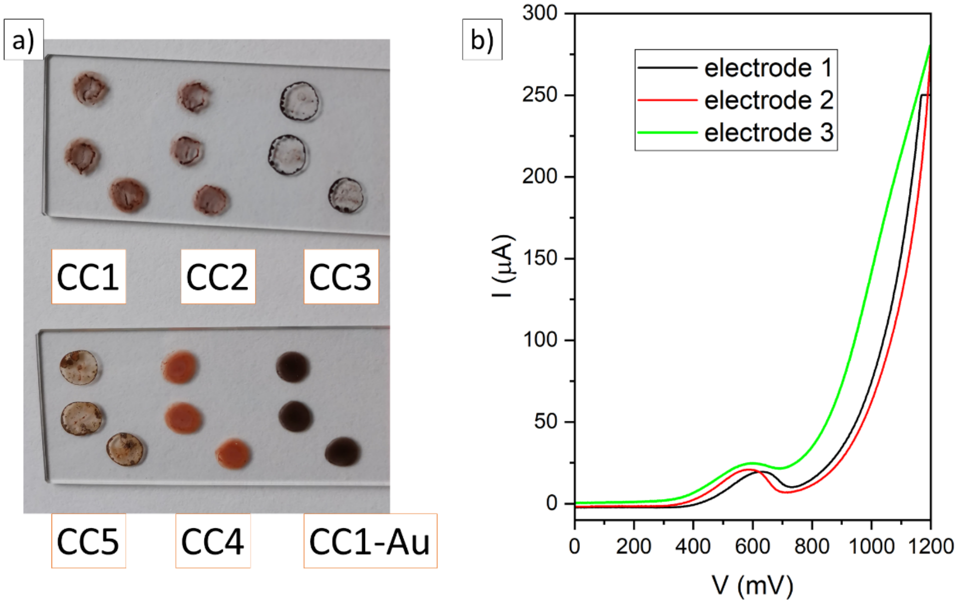

Figure S1. (a) Photographs of prepared pastes drops displaying powder dispersion in the detection layer, (b) cyclic voltammetry tests proving the reproducibility of the electrodes (paste based on the CC1).

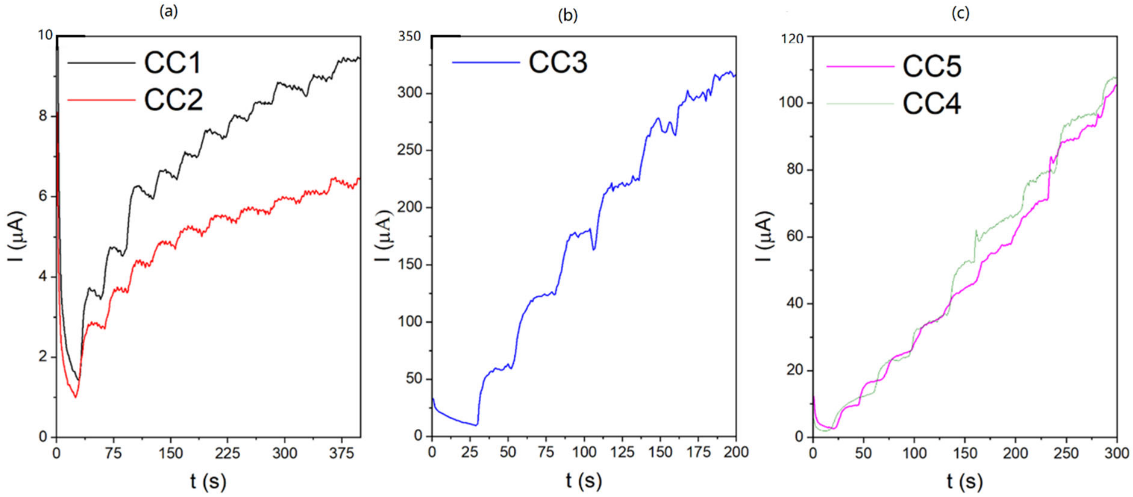

Figure S2. Amperometric response of the copper oxide modified GCE electrodes recorded at the applied potential of the (a) 660 mV, (b) 650 mV, and (c) 640 mV.
